# Potassium Linoleate (Isomerized) Satisfies the United States Environmental Protection Agency MB-05-16 for Hospital Disinfectant on Hard, Non-porous Surfaces

**DOI:** 10.7759/cureus.22851

**Published:** 2022-03-04

**Authors:** David G Changaris, Anne L Carenbauer

**Affiliations:** 1 Microbiology, Ceela Naturals, LLC, Louisville, USA

**Keywords:** hospital-acquired infection, cosmetic cleanser, cross-kingdom antimicrobial, cla antibacterial, surface disinfectant, mb-05-16, potassium linoleate (isomerized), potassium linoleate (conjugated)

## Abstract

Potassium conjugated linoleic acid or potassium linoleate (isomerized), 86 mM, satisfies the United States Environmental Protection Agency protocol hospital disinfectant for non-porous surfaces MB-05-16 with one-minute treatment. This stringent protocol requires separate preparations of *Staphylococcus aureus* (American Type Culture Collection 6538) and *Pseudomonas aeruginosa *(American Type Culture Collection 15442) unstirred for 48 hours, submerging 10 mm polished cylinders in the culture, and placing for 45 minutes in a 37°C humidified chamber before treating. Since potassium linoleate (isomerized) also satisfies the United States Environmental Protection Agency protocol MB-35-00 for *Candida auris*, this study establishes potassium linoleate (isomerized) as an effective cross-kingdom antimicrobial plant salt, soap, or cleanser. We affirm the need for formal post-treatment plating on agar to establish efficacy and not rely on OD_600 _when testing for antimicrobial capacity. Aqueous dilution of the soap causes variable opalescence making optical density an unreliable marker for antimicrobial efficacy.

## Introduction

Surface disinfection has moved center stage in efforts to reduce hospital-acquired infections (HAI) [1-4]. The observed correlation between drug-resistant bacteria within a hospitalized patient and subsequent infection in another patient who subsequently occupies the same room has increased “turn-over room disinfection” efforts with some success [5-8].

Efforts to reduce the transmission of drug-resistant microbes to the next occupant of the room include quaternary amines, bleach, peroxide, peracetic acid, ultraviolet light (ozone), aerosolized disinfectants [7]. Exposing hospital personnel to these disinfectants can cause skin and pulmonary injuries [9-11]. These increasingly toxic procedures require longer recovery times before humans can enter an increasingly exotic biosphere. So far none of these efforts has resulted in eliminating HAI.

Of late, we have learned that fungi serve as reservoirs for lateral-gene transfer of antimicrobial resistance genes [12]. And we know that in some settings, reducing the complexity of the biosphere enhances antimicrobial resistance [13-15]. Ironically, the fundamental goal to disinfect a room may fail because it seeks to eliminate microbial concentrations. Such biocidal activity, over time in many settings, will lead to resistance formation.

In conjugated linoleic acid, the diene confers the oil with water miscibility and the capacity to form emulsions in the presence of amino acids and peptides [16]. These C-18 conjugated diene salts, in high concentrations, have broad-spectrum lethality to Gram-positive and Gram-negative bacteria, yeast, and fungi. The reported antimicrobial efficacy included antibiotic-resistant bacteria such as methicillin-resistant staphylococcus and vancomycin-resistant enterococcus [17,18]. The recent article noting efficacy on *Candida auris* with a broad antimicrobial resistance spectrum adds another level of efficacy. Potassium linoleate (conjugated) may be applied to both environment and human skin, airway, and gut [19]. It is part of our human physiology. In this context, this may offer novel approaches to HAI prevention treating the room as well as patient, concurrently.

## Materials and methods

Environmental considerations for potassium linoleate (isomerized) and diluents

**Figure 1:**
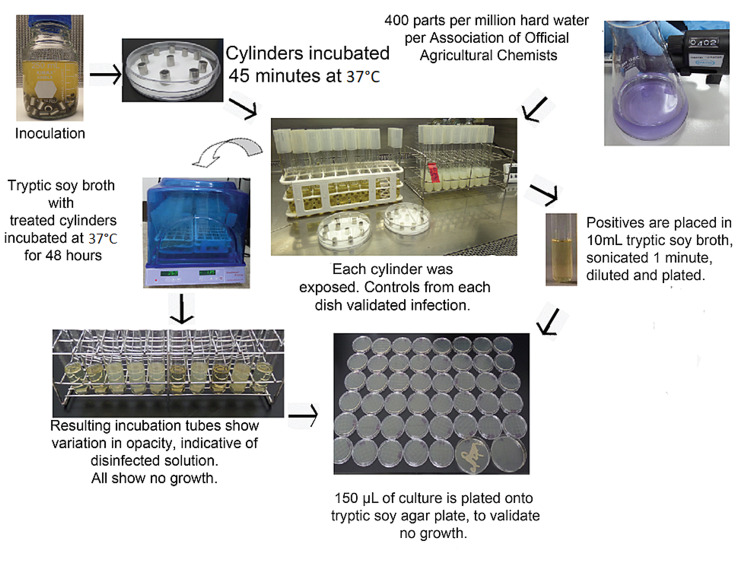
General outline of United States Environmental Protection Agency protocol for hospital disinfection MB-05-16. Hard water was prepared per American Official Agricultural Chemists (AOAC); Tryptic Soy Broth (TSB) was prepared fresh.

Conjugated linoleic acid (CLA), purchased from Innobio, Dallan, China, has generally recognized as safe (GRAS) food status by the United States Food and Drug Administration Center for Food Safety and Applied Nutrition [20]. CLA is considered environmentally safe [21,22]. It does not require a neutralization agent for disposal. Abundant naturally occurring enzymes participate in degradation as well as natural oxidative stressors metabolize CLA [20,21]. For this reason, neutralization efficacy tests were performed once, and validated, as our “neutralizing agent” in all cases is the preferred growth media of the organism tested. The outline for the United States Environmental Protection Agency (US EPA) MB-05-16 is shown in Figure 1.

MB-10-07 (the United States Environmental Protection Agency, September 27, 2019)

General maintenance standard operating procedures (SOP) for the microbiology lab with respect to the good lab practice for the generation of media and reagents. It specifies how to wash glassware and generate common media and agars for a Petri-plate generation. The SOP also deals with the handling of microbes on agar and in culture, common things to inspect before using, and other good lab practices for microbiology facilities.

MB-22-05 (the United States Environmental Protection Agency, May 22, 2019b)

This SOP defines dilution procedures for the test substances, formerly QC-22-04. Time limitations on using dilutions, how to generate test spray bottles, and similar specifics are noted here. Parameters defined in this SOP were incorporated into the protocols used in experimentation.

MB-03-07 (the United States Environmental Protection Agency, July 3, 2017)

The screening method was used to test the cylinders. Since we are testing these soaps as surface sanitizers, we tested the potassium linoleate (isomerized) to qualify the cylinders. Penicylinders were cut from 316 stainless steel tubings (Elmhurst, IL: McMaster-Carr Supply Company), 8mm OD, 6mm ID into 10 mm lengths. These were then polished in a vibrating polisher for one week each on three various resin polishes, coarse, fine, and pre-polish. The cylinders and polish resin were allowed to run for two weeks. After this time, the cylinders were sonicated in a mild detergent to remove the fine metal and polish powder. They were then prepared with a 1.0 M sodium hydroxide, washing overnight, followed by distilled water rinsing, the final rinse checked with 1% phenolphthalein indicator solution (Hatfield, AR: Biopharm Inc.) to be clear of the pink and residual base.

MB-30-02 (the United States Environmental Protection Agency August 21, 2019)

Hard water was generated per the Association of Official Agricultural Chemists protocol MB-30-02 and was used to dilute test substances. Magnesium chloride, anhydrous calcium chloride, and sodium bicarbonate were obtained from Carolina Biological Supply Company. Stock solutions A and B were generated with purified water and were subsequently sterilized with a 0.1μm membrane filter within a 250 mL VWR® Vacuum Filtration System (Radnor, PA: VWR International). After mixing, the water was titrated using the Hach® 5B Water Hardness Test Kit (Loveland, CO: Hach Company) using the 0.800 M ethylenediaminetetraacetic acid cartridge. Hard-water titrations resulted in water between 382 and 403 ppm total hardness, depending on the date of use. Filtration for sterilization followed.

MB-05-16 (formerly MB-05-14; the United States Environmental Protection Agency, May 16, 2020)

Association of Official Agricultural Chemists (AOAC) use dilution test (AOAC 955.14; 955.15; 964.02) has a general outline of the method shown in Figure 1. In this method, a 48-hour unshaken culture infects 10 mm steel cylinders. The cylinders, after 15 minutes of infection, are incubated for 45 minutes at 37ºC to dry. The cylinders are then individually treated with the diluted test solution and transferred to recovery media, tryptic soy broth. Per the SOP, the maximum contact time is 10 minutes. One minute incubation time was used throughout our testing. The recovery media was allowed to incubate for 48 hours, and the culture tubes were assessed. For our experiments, all culture tube contents at 48 hours were plated to a tryptic-soy-agar plate. All materials were brought to room temperature (69ºF) prior to use. To minimize possible contamination, all work was performed in the Envirco® Class II Biohazard Cabinet (Albuquerque, NM: Envirco Corporation).

Phosphate Buffered Saline

Phosphate buffered saline (PBS) was generated in-house using basic salts. Sodium chloride was obtained from Aldon Corporation (Avon, NY). Potassium chloride was obtained from Ingredi Company (Baltimore, MD). Sodium phosphate dibasic, anhydrous, and potassium phosphate monobasic were obtained from Carolina Biological Supply Company (Burlington, NC). 

Growth Media and Agars

Tryptone glucose extract agar was obtained from Himedia™ (Mumbai, India: HiMedia Laboratories Pvt Ltd Company), trypticase soy broth from Beckton-Dickinson™ (Franklin Lakes, NJ: Becton, Dickinson and Company), tryptic soy agar from Criterion™ (Santa Maria, CA: Hardy Diagnostics Company), and Sabouraud dextrose agar and Sabouraud dextrose broth were obtained from Weber Scientific Inc. (Hamilton Township, NJ). Letheen broth base was used in combination with Tween™ 80 (Salon, OH: MP Biomedicals). Cetrimide agar from Himedia™ with glycerol from Spectrum Chemicals (New Brunswick, NJ: Spectrum Chemical Mfg Corporation) was used to validate pseudomonas recovery. CHROMagar pseudomonas and CHROMagar candida from CHROMagar were used to validate phenotypes. All media were prepared per label instructions, using purified water. pH was checked using a Hanna pH meter (Woonsocket, RI: Hanna Instruments Inc.), and adjustments were made using either 1.0 M hydrochloric acid or 1.0 M sodium hydroxide. Sterilization was performed using the Tuttnauer EZ-10 autoclave (Hauppauge, NY: Tuttnauer USA) unless instructions were to boil. All Petri dishes were obtained pre-sterilized and ready for use from various suppliers; 100 mm x 15 mm dishes were used for direct plating and 65 mm x 15 mm were used to incubate membrane filters.

Bacterial Strains

Bacterial strains required for testing were obtained from the American Type Culture Collection (ATCC) in Atlanta, GA. *Staphylococcus aureus* ATCC 6538, and *Pseudomonas aeruginosa* ATCC 15442 were received in frozen form and were recovered per method recommendations for each strain. Glycerol stocks were generated and stored at -80°C. Recovery and responsiveness to CHROMagar and selective media were confirmed. Plate-to-plate transfers were limited to five passages and then discarded. A new glycerol stock was then thawed, plated, and used for testing. A Scientemp© -80ºC freezer (Adrian, MI: Scientemp Corporation) maintained the stocks. All work was performed in an Envirco bio-safety cabinet (Model 10274), certified by Steris (OH, USA). Southwest Science Mini IncuShaker™ (model SH1000; Santa Fe, NM: Southwest Sciences Inc.) was used for incubation.

Purified Water

Local water was filtered through carbon and deionizing tanks before reverse osmosis filtration. The water was passed through two reverse osmosis systems and two ultraviolet treatment protocols. The conductivity and total organic carbon are monitored. The water is stored in a 316-liter stainless steel container at 180-185°F prior to use. The purified water passed endotoxin testing and total organic carbon (TOC) before and after conducting experiments. The resulting water also satisfies the standards for purified bulk water <USP645>.

## Results

**Figure 2:**
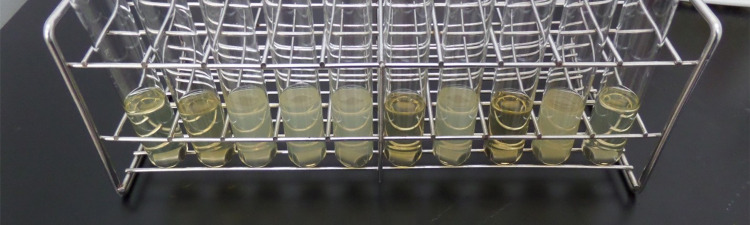
Variation of the opacity of media from trials. All tubes were verified “no growth” with plating.

Three separate lots of potassium linoleate (Isomerized) fulfilled the United States Environmental Protection Agency protocol MB-05-16 with each assay showing 0 or 1 of 60-85 cylinders remaining culture-positive after 69-86 mM exposure for one minute (Table 1). This plant-oil salt satisfies EPA protocols for hospital disinfection on nonporous surfaces at 86 mM with one minute contact time. The EPA protocols define the need to perform the entire testing on three separate manufactured lots. Each of the lots were manufactured on separate days. Additionally, one of the lots must be older than 60 days from the time of manufacture. Lot 1 was tested more than 60 days after manufacture. The results have been shown in Table 1. Figure 2 shows the wide variation in opacity despite all tubes having negative growth.

**Table 1:** Potassium linoleate (isomerized) disinfects polished 316 stainless steel tubes. Results from disinfecting steel cylinders exposed to pseudomonas and staphylococcus with 69-86 mM potassium linoleate (isomerized). ATCC: American Type Culture Collection

Batch	mM	ATCC	# Cylinders Growth Positive	Pass/Fail
1	69	*Staphylococcus aureus *6538	0/85	Pass
2	86	*Staphylococcus aureus* 6538	0/60	Pass
3	86	*Staphylococcus aureus* 6538	0/60	Pass
1	86	*Pseudomonas aeruginosa* 15442	0/72	Pass
2	86	*Pseudomonas aeruginosa *15442	1/60	Pass
3	86	*Pseudomonas aeruginosa* 15442	0/60	Pass

**Figure 3:**
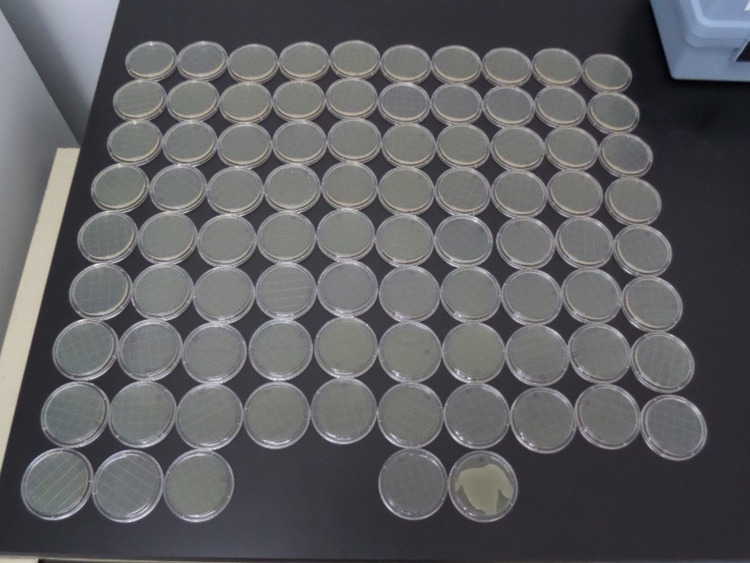
Pseudomonas aeruginosa American Type Culture Collection 15442 treated cylinders exposed to 86 mM potassium linoleate (isomerized) shows no growth except control (bottom right).

Figure 3 shows the absence of growth in the Petri dishes from batch 1, *P. aeruginosa* American Type Culture Collection 15442 treatment. Similar visual documentation is available for all trials for this study per the United States Environmental Protection Agency protocol MB-05-16. 

## Discussion

The qualification protocol for qualifying stainless steel penicylinders (US EPA protocol MB-03-07) incorporates the use of BTC® 835 (Maywood, NJ: Stepan). This disinfectant is composed of alkyl-dimethyl benzyl ammonium chloride, water, ethanol, and alkyl-dimethyl amines. Faced with the prospect of changing the hazmat status of the entire building with available respiratory protection protocols and training, we chose to qualify the cylinders with potassium linoleate (isomerized).

This hesitancy to expose working personnel to quaternary amines herein illustrates the complex choices daycare and food establishments face. Quaternary amines, while effective, require environmental hazard protocols that pose added cost and risk. Potassium linoleate (isomerized) fulfills the United States Environmental Protection Agency protocol MB-05-16 and offers, potentially, a safer and equally effective hospital-grade disinfectant. The concentration used within this test falls within the range currently sold as a cosmetic cleanser. Anecdotal reports of this cosmetic cleanser providing the appearance of antimicrobial efficacy exist. There are no specific limitations for contact with humans, neutralization agent, harmful vapors, or residuals. This may permit novel testing protocols where the same disinfectant used for environmental disinfection could offer direct prophylactic patient exposure to limit HAI.

Technically, *Candida albicans and Candida auris *belong to the eukaryote kingdom - Fungi [23]. We have published elsewhere that potassium linoleate (isomerized) has the capacity to rapidly kill these two yeast/fungi [24]. With the present work, we establish that potassium linoleate (isomerized) has microbiocidal activity against Staphylococcus classified as a member of the kingdom Bacteria, subkingdom Posibacteria [25], and against Pseudomonas, which is also a member of the kingdom Bacteria but within the subkingdom Negibacteria [26]. This defines potassium linoleate (isomerized) as having cross-kingdom microbiocidal activity.

As shown in Figure 2, aqueous dilutions of potassium linoleate (isomerized) in hard-water or nutrient broth become inconsistently cloudy. This makes optical density ineffective at screening growth. Perhaps this simple fact has contributed to the delay in recognizing the broad-spectrum cross-Kingdom-antimicrobial capacity of potassium linoleate (isomerized). This report belies the 1992 EPA ruling that all cationic plant oil salts do not possess microbiocidal activity [27].

The narrow scope of this study defines its limitations. The protocols used are robust for the narrow purpose of identifying significant surface disinfection efficacy as defined by the US EPA. This study does not identify mechanisms of disinfection efficacy nor does it address the prospect of slime formation to limit efficacy. Moreover, this study has not been subjected to regulatory approval that will be required before it can be introduced to interstate commerce.

## Conclusions

Based upon our past studies, one consistent observation endures, namely, potassium linoleate (isomerized) provides rapid disinfection of surfaces. We also note that *in vivo* anecdotal reports from its cosmetic use have clearly identified potential clinical microbiocidal relevance. This plant oil salt satisfies EPA protocols for hospital disinfection on nonporous surfaces at 86 mM with one minute contact time. This includes the one lot of potassium linoleate (isomerized) being older than 60 days. In general, potassium linoleate (isomerized) has potential as an antimicrobial agent that will require regulatory approval before introduction to interstate commerce.
